# Usefulness of FDG PET/CT in the management of tuberculosis

**DOI:** 10.1371/journal.pone.0221516

**Published:** 2019-08-27

**Authors:** Adrián Sánchez-Montalvá, Marta Barios, Fernando Salvador, Ana Villar, Teresa Tórtola, Daniel Molina-Morant, Carles Lorenzo-Bosquet, Juan Espinosa-Pereiro, Israel Molina

**Affiliations:** 1 Infectious Diseases Department, Vall d’Hebron University Hospital, PROSICS Barcelona, Universitat Autònoma de Barcelona, Barcelona, Spain; 2 Grupo de Estudio de micobacterias (GEIM), Sociedad Española de Enfermedades Infecciosas y Microbiología Clínica (SEIMC), Madrid, Spain; 3 Nuclear Medicine Department, Vall d’Hebron University Hospital, Universitat Autònoma de Barcelona, Barcelona, Spain; 4 Pneumology Department, Vall d’Hebron University Hospital, Universitat Autònoma de Barcelona, Barcelona, Spain; 5 Microbiology Department, Vall d’Hebron University Hospital, PROSICS Barcelona, Universitat Autònoma de Barcelona, Barcelona, Spain; University Tor Vergata, ITALY

## Abstract

**Background:**

The aim of our study is to describe the FDG-PET/CT findings in patients with tuberculosis and to correlate them with the patient’s prognosis.

**Methods:**

We retrospectively collected data from patients with tuberculosis, who had an FDG-PET/CT performed prior to treatment initiation from 2010 to 2015.

**Results:**

Forty-seven out of 504 patients with active tuberculosis diagnosis (9.33%) underwent an FDG-PET/CT. The reasons for performing the FDG-PET/CT were: characterization of a pulmonary nodule (24; 51.1%), study of fever of unknown origin (12; 25.5%), study of lymph node enlargement (5; 10.6%) and others (6; 12.8%). Median age was 64 (IQR 50–74) years and 31 (66%) patients were male. Twenty-six (55.3%) patients had an immunosuppressant condition. According to the FDG-PET/CT, 48.6% of the patients had more than 1 organ affected and 46.8% had lymph node involvement. Median SUVmax of the main lesion was 5 (IQR 0.28–11.85). We found an association between the FDG accumulation and the size of the main lesion with a correlation coefficient of 0.54 (p<0.002). Patients with an unsuccessful outcome had a higher ratio SUVmax main lesion / SUVmean liver (1.92 vs 7.67, p<0.02).

**Conclusions:**

In our cohort, almost half of the patients had more than 1 organ affected and 46.8% of them had lymph node involvement. FDG uptake was associated with the size of the main lesion and seems to be related to the treatment outcome. The extent of its potential to be used as an early predictor of treatment success still needs to be defined.

## Introduction

Tuberculosis (TB) remains as one of the leading killers of our time. According to the World Health Organization, in 2015 there were an estimated 10.4 million new cases and 1.8 million people died from TB worldwide [1]. In high-income countries, TB has decreased abruptly in the last century, with incidence dropping below 40 cases per 100.000 inhabitants. At the present time, TB in low incidence countries is mainly diagnosed in migrants coming from high incidence countries or in immunosuppressed patients [2].

TB is suspected based on epidemiology, symptoms and additional tests [3]. However, many patients may present unusual symptoms and signs, hindering TB diagnosis. Moreover, in low incidence countries the positive predictive value of these symptoms is undermined by the low prevalence of the disease. Hence, TB is not always among the initial diagnosis in patients with the aforementioned symptoms.

Molecular imaging based on positron emission tomography (PET) with glucose analogue 2-[fluorine 18]fluoro-2-deoxy-D-glucose (FDG) has been extensively used in oncologic disease, as it refines the staging of the disease and allows for an oncologic burden analysis that has shown to be more accurate in some tumours than at the clinical stage [4]. Uses outside the oncologic field are also being explored, mainly in inflammatory and infectious diseases [5]. Enhanced activation of the immune system cells boosts the cell glycolysis, even in the presence of immunosuppression. Consequently, the uptake of FDG is increased, resulting in a higher standardized uptake value (SUV) measurement [6]. Furthermore, many experts include the FDG PET/CT in the study algorithm of a fever of unknown origin and in the characterization of pulmonary nodules [7,8].

FDG PET/CT has been scarcely reported in patients with TB in low incidence TB countries. Active TB lesions have an increased FDG uptake, regardless of the location of the lesion [9]. The burden of disease in a patient with pulmonary TB is measured using conventional X-ray and microbiologic parameters such as bacilli counting in positive sputum smear patients or time to positive culture. However, the real extension of the disease may go unnoticed with the limited information provided on the activity of the disease or other affected organs. More recently, it has been hypothesized that FDG PET/CT may assist in the evaluation of the response to the treatment [10–13].

The aim of our study is to describe the FDG PET/CT findings in patients with pulmonary and extrapulmonary TB in a cohort of patients with a high proportion of immunosuppressed patients from a low incidence TB country. The secondary objective is to correlate the findings of the FDG-PET/CT with the prognosis of the disease.

## Methods

### Study population

An observational retrospective study was conducted at the Vall d’Hebron University Hospital (VHUH). All patients diagnosed with TB from May 2010 to May 2015 were reviewed. We selected adult patients who had an FDG PET/CT performed within the two months prior to the initiation of the antituberculous treatment. The decision to perform an FDG PET/CT was at the discretion of the treating physician. Data regarding tuberculosis variables were retrieved from the medical records.

### Tuberculosis diagnosis

Patients were considered to have active tuberculosis if they had symptoms and a specimen positive for *M*. *tuberculosis* either in a specific culture or in a molecular biology test (GenXpert MTB/RIF^®^, Cepheid, Sunnyvale, CA). Patients with symptoms, a positive histology, an epidemiological background and a favourable response to treatment were also considered to have TB. Treatment with antituberculous drugs was initiated immediately after the diagnosis or the clinical suspicion. The therapy regimen and the duration of the treatment were decided by the treating physician. The final TB outcome was recorded following the WHO recommendations [14].

### PET imaging

All patients were required to fast for 4 hours prior to PET/CT scanning to achieve blood glucose concentrations below 150mg/dL. Images were obtained from the skull base to the proximal third thigh one hour after ^18^F-FDG intravenous administration of about 3.7 MBq/Kg. We used two different PET/CT machines, Scanner 1 (PET/CT Siemens Biograph 6) and Scanner 2 (PET/CT Siemens Biograph mCT). An iodine-based intravenous contrast agent was also used. All images were evaluated by two medical experts in nuclear medicine who were blinded to clinical information.

The PET analysis was conducted based on quantitative analysis. More concretely, maximal standardized uptake value (SUVmax) was obtained for pathological lesions at any location, including lymph nodes, by manually delineating the affected area on attenuation-corrected axial images. The averaged value was used in case of positive adjacent lesions. Measurements and characteristics of the lesions were assessed in the CT images and when adjacent lesions were present an averaged measurement was used. The size was used to define the main TB-related lesion. Unclear cases were defined on agreement between medical experts in nuclear medicine and infectious diseases experts. We summed the SUVmax of all TB-related lesions to get SUV total, as a surrogate marker of disease burden. Lymph node basin included cervical, hilar, mediastinal, axillary, portal hepatic, paraaortic, pelvic and inguinal regions. SUVmax and SUVtotal were divided by liver mean SUV to obtain a standardized-quotient regardless of the scanner used to acquire the images (ratio SUVmax/SUVliver and ratio SUVtotal/SUVliver). In patients with malignancies, the FDG PET/CT parameters were determined according to the histologic results obtained from the main lesion and the lymph nodes, and only TB-related lesions were included in the analysis.

### Compliance with ethical standards

The study protocol was approved by the Ethical Review Boards of Vall d’Hebron University Hospital (Barcelona, Spain). An exemption from obtaining informed consents was granted. Procedures were performed in accordance with the ethical standards laid down in the Declaration of Helsinki as revised in 2013.

### Statistical analysis

The medians and interquartile ranges (IQR) or the means and standard deviations (according to the distribution) were calculated for quantitative variables. Frequencies and percentages were calculated for qualitative variables. The analysis was performed using Student’s *t*-test or Mann–Whitney’s *U* test for quantitative variables and Chi-square test or Fisher’s test for qualitative variables when appropriate. PET/CT-adjusted analysis using a logistic regression model was performed to correct the SUV difference between scanners. Tests were considered significant when the two-tailed *p*-value was <0.05.

## Results

During the studied period, 504 patients were diagnosed with TB at our institution. Forty-seven (9.33%) patients had an FDG PET/CT. The reasons for performing the FDG-PET/CT were characterization of pulmonary nodule (24; 51.1%), study of fever of unknown origin (12; 25.5%), study of lymph node enlargement (5; 10.6%) and others (6; 12.8%), including 3 patients with cachexia, asthenia and anorexia, one patient with pulmonary infiltrate, one patients with secondary amyloidosis and one patient with pleural effusion. The median age of the studied population was 64 (IQR 50–74) years and 31 (66%) patients were male. More than half of the cohort (55.3%) had an immunosuppressant condition. Scanner 1 was used in 17 (36.2%) patients and Scanner 2 in 30 (63.8%) patients. More information about clinical and epidemiological data of the cohort is shown in Table 1.

**Table 1:** Epidemiologic and clinical features of the cohort (n = 47).

Sex, male	31 (66%)
Age, years	64.04 (50.33–74.35)
Nationality, Spanish	37 (78.7%)
Smoking	21 (44.7%)
Inmunosuppression condition	26 (55.3%)
*Concomitant solid tumor*	10 (38.5%)
*Transplantation*	4 (15.4%)
*Autoimmune disease*	5 (19.2%)
*Hematological condition*	3 (11.5%)
*HIV*	1 (3.8%)
*Others*	3 (11.5%)
Chronic lung disease	17 (36.2%)
Diabetes Mellitus	10 (21.3%)
Chronic renal impairment	9 (19.1%)
Previous TB disease	6 (12.8%)
Chronic liver disease	4 (8.5%)

HIV: human immunodeficiency virus. TB: tuberculosis

Regarding clinical TB data, 28 (59.6%) patients were diagnosed with lung TB. Thirty-seven (78.7%) cases were microbiologically confirmed TB. Treatment length was 6 month in 28 patients (70% of patients who finished the treatment). A successful outcome was achieved in 85.1% of the patients. For further information see Table 2.

**Table 2:** Clinical TB data.

Symptoms	
*Fever*	19 (40.4%)
*Cough*	16 (34%)
*Sweating*	10 (21.3%)
*Loss of weight*	13 (27.7%)
Hospitalization	31 (66%)
Type of Tuberculosis	
*Lung*	28 (59.6%)
*Extrapulmonar*	13 (27.7%)
*Mixed*	6 (12.8%)
Tuberculosis outcome	
*Treatment success*	40 (85.1%)
*Transfer out*	1 (2.1%)
*TB related death*	2 (4.3%)
*Non-TB related death*	4 (8.5%)
*Treatment failure or recurrence*	0
Tuberculosis diagnosis	
*Microbiologically confirmed*	37 (78.7%)
*Histologically compatible*	7 (14.9%)
*Clinical suspicion*	3 (6.4%)
Resistance	
*No resistance*	27 (59.6%)
*Monoresistance*	3 (4.3%)
*Multiresistance non-MDR*	1 (2.1%)
*Not performed*	1 (2.1%)
Intensive therapy	
*Isoniazid*	44 (93.6%)
*Rifampicin*	41 (87.2%)
*Pirazinamid*	40 (85.1%)
*Ethambutol*	34 (72.3%)
*Quinolones*	6 (12.8%)
*Aminoglucosydes*	1 (2.1%)
Maintenance therapy	
*Isoniazid*	41 (87.2%)
*Rifampicin*	39 (82.9%)
*Quinolones*	4 (8.5%)
Total length of the regimen (months) n = 40	6 (6–8)
≤6 months (percentage)	28 (70%)
>6–12 months (percentage)	7 (17.5%)
≥12months (percentage)	5 (12.5%)

TB: tuberculosis, MDR: multidrug resistant tuberculosis

Findings from the FDG PET/CT are summarized in Table 3. It is worth noting that 48.7% of the patients had more than 1 organ affected and 46.8% of the patients had lymph node involvement. Median SUVmax of the main affected lesion was 5 (IQR 1.28–11.58). As for the lymph node, the median SUVmax of the biggest affected lymph node was 11.7 (6.8–19.4). Patients with immunosuppression did not show higher uptake of FDG in the ratio SUVtotal divided by SUVmean of the liver compared to non-immunosuppressed patients (11.37 vs 8.65; p = 0.471). However, SUV values in immunosuppressed patients tend to be higher. When comparing other PET/CT parameters according to immunosuppression condition, we did not find any statistically significant difference.

**Table 3:** FDG-PET/CT findings.

Reason to perform FDG-PET/CT	Overall (n = 47)
*Pulmonary nodule*	24 (51.1%)
*Fever of unknown origin*	12 (25.5%)
*Enlarge lymph nodes*	5 (10.6%)
*Others*	6 (12.8%)
Main affected organ	
Lung	25 (53.2%)
Lymph node	8 (17%)
Pleura and/or pericardium	5 (10.6%)
Psoas abscess	1 (2.1%)
Pneumonectomy cavity	1 (2.1%)
CNS	1 (2.1%)
Inactive lesions*	6 (12.8%)
Number of metabolic lesions in the main organ	
*1*	16/37 (43.2%)
*2*	7/37 (18.9%)
*>2*	14/37 (37.8%)
Median SUVmax main lesion	5 (1.28–11.85)
Mean SUVmean liver	2.2 (0.76)
Median ratio SUVmax main lesion/ SUVmean liver	2.1 (0.56–4.9)
Mean SUVmax liver	3.34 (1.13)
Median ratio SUVmax main lesion/ SUVmax liver	1.4 (0.37–3.17)
Median of the highest diameter main lesion (mm) (N = 31)	20 (16–34)
Number of affected organs	
*1 affected organ*	20/39 (51.3%)
*2 affected organs*	16/39 (41%)
*≥3 affected organs*	3/39 (7.6%)
Lymph node involvement	22 (46.8%)
Number of metabolic lymph node basin	
*None affected basin*	25 (53.2%)
*1 affected basin*	5 (10.6%)
*2 affected basin*	4 (8.5%)
*3 affected basin*	3 (6.4%)
*4 affected basin*	3 (6.4%)
*≥5 affected basin*	7 (14.9%)
Median diameter of biggest lymph node (mm)	21 (17–33.5)
Median SUVmax of the biggest lymph node	11.7 (6.8–19.4)
Median SUVtotal	8.4 (1.7–28.7)
Median SUVtotal/SUVmean liver	4.31 (0.99–14.37)

Note:

*5 patients had FDG uptake due to cancer lesions with histology confirmation. One patient with tuberculous peritonitis did not show FDG uptake. SUV: standardized uptake value.

**Fig 1:**
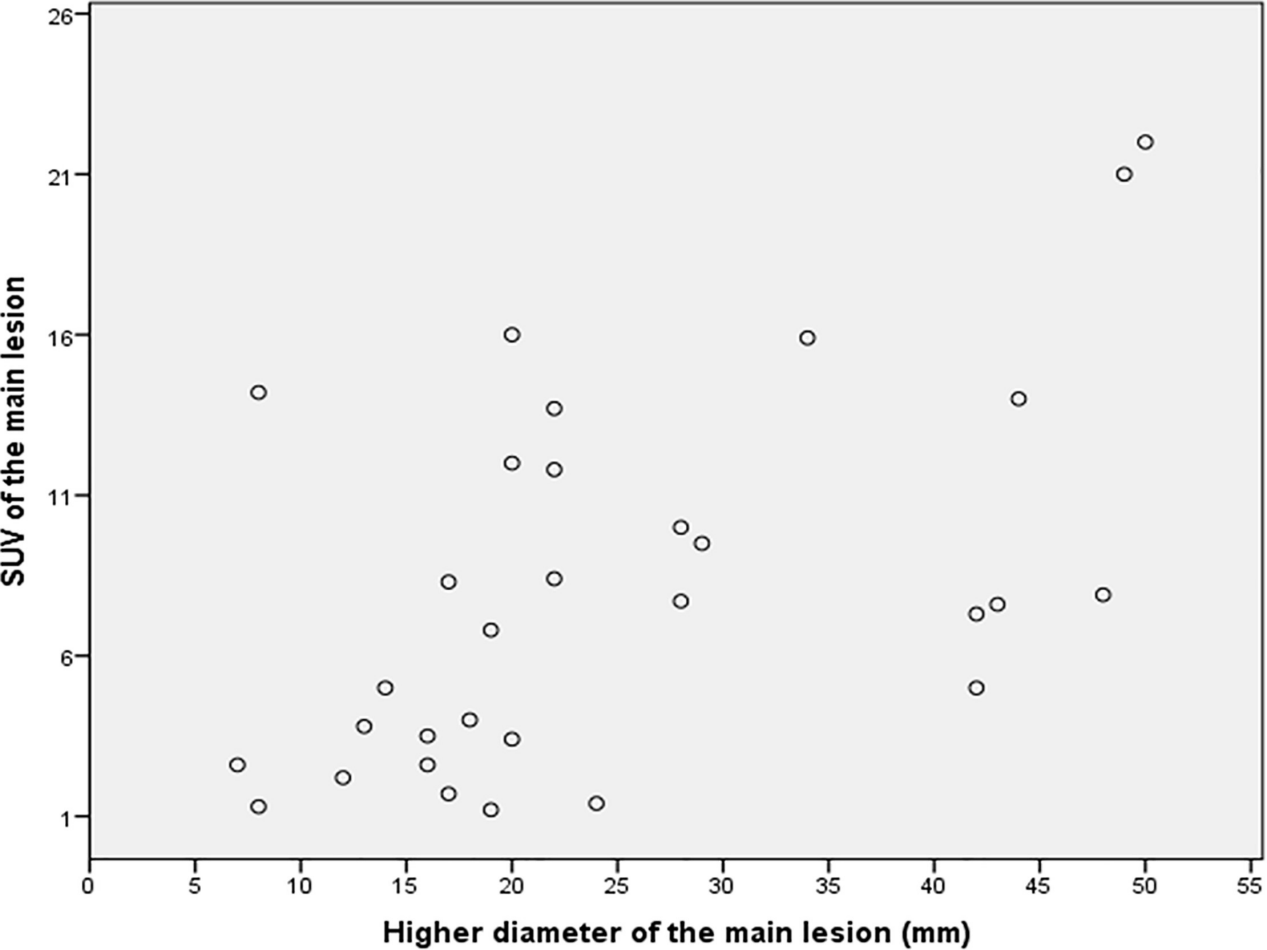
Scatter diagram paring higher diameter of the main lesion (mm) and SUV of the main lesion.

We found an association between the FDG accumulation and the size of the main lesion with a correlation coefficient of 0.54 (p<0.002). Fig 1 shows a scatter diagram pairing size of the main lesion and uptake of FDG. SUVmax of the main lesion, ratio SUVmax of the main lesion / SUV mean liver and ratio SUVtotal / SUV mean liver were correlated with the number of affected organs, and with the presence of affected lymph node basins (Table 4).

**Table 4:** Relationship between size and FDG accumulation and disease burden.

	1 organ affected	>1 organ affected	P value
Size main lesion (mm)	19.5 (15.5–25)	28 (17.5–45)	0.378
SUVmax main lesion	3.15 (1.22–8.15)	9.25 (3.87–12.75)	**0.035**
Ratio SUVmain lesion / SUVmean liver	1.62 (0.49–4.31)	3.8 (1.2–6.8)	**0.035**
Ratio SUVtotal/ SUVmean liver	1.62 (0.49–4.6)	15.51 (5.9–29.43)	**<0.001**
	Non-affected lymph node	Affected lymph node	P value
Size main lesion (mm)	19 (14–24)	25 (17.25–42)	0.59
SUVmax main lesion	2.6 (0–7.4)	9.25 (3.87–14.4)	**0.002**
Ration SUVmain lesion / SUV mean liver	1.08 (0–3.43)	4.26 (1.82–7.03)	**0.002**
Ratio SUVtotal/ SUVmean liver	1.18 (0–3.48)	15.61 (8.4–28.5)	**<0.001**

Values are median and interquartile range (U Mann-Whitney test).

When comparing treatment length, hospitalization stay and TB outcome with FDG PET/CT findings (Table 5), we found that patients with an unsuccessful outcome had higher FDG uptake in the main lesion and higher ratio SUV main lesion / SUV mean liver. We constructed a receiver operating characteristic (ROC) curve for the ratio SUVmax in the main lesion divided by SUV mean of the liver to identify an unsuccessful outcome that rendered an area under the curve at 0.812 (SD 0.159; 95%CI 0.502–1; p = 0.024). A ratio value of 7.4 gives a sensitivity and specificity for an unsuccessful outcome of 0.8 and 0.976, respectively.

**Table 5:** Treatment length, hospitalization and cure according to PET/CT parameters.

	Treatment 6 months	Treatment >6 months	p value	Machine-Adjusted p value*	OR 95CI
SUVmax main lesion	3.15 (0.25–7.85)	8.65 (2.95–11.95)	**0.011**	**0.015**	**1.26 (1.05–1.52)**
Size (mm)	20 (13.5–28)	19 (17–25.5)	0.683		
SUV total	4.15 (0.3–19.93)	24.45 (16.65–77.52)	**0.002**	**0.005**	**1.06 (1.02–1.1)**
Affected organs = 1	17 (65.4%)	4 (33.3%)	0.065		
Affected basin ≤2	22 (78.6%)	6 (50%)	0.071		
Ratio SUV main lesion / SUV mean liver	1.5 (0.11–4.07)	3.4 (1.26–4.98)	0.130		
Ratio SUVtotal /SUV mean liver	2.15 (0.11–9.05)	11.06 (2.5–29.43)	**0.011**	**0.006**	**1.12 (1.03–1.22)**
	Hospitalization	Outpatient office	p value	Machine-Adjusted p value*	OR 95CI
SUVmax main lesion	7.3 (0–12)	2.75 (1.22–2.75)	0.22		
Size (mm)	25.5 (17–43.3)	22 (16.8–28)	0.894		
SUV total	21.3 (2.6–52.5)	3.95 (1.33–8.23)	**0.028**	**0.023**	**1.06 (1.01–1.12)**
Affected organs = 1	13 (44.8%)	12 (75%)	0.051	**0.034**	**4.7 4 (1.13–19.89)**
Affected basin ≤2	20 (64.5%)	14 (87.5%)	0.095		
Ratio SUV main lesion / SUV mean liver	3.17 (0–6.74)	1.28 (0.67–4.07)	0.237		
Ratio SUV total /SUV mean liver	8.85 (1.20–25.92)	1.91 (0.66–4.14)	**0.041**	**0.028**	**1.1 (1.01–1.20)**
	Cure	Dead	p value	Machine-Adjusted p value*	OR 95CI
SUVmax main lesión	4 (1.25–925)	14.2 (7–18.95)	**0.042**	**0.035**	**1.22 (1.02–1.47)**
Size (mm)	20 (16–28)	39(14.5–48.5)	0.545		
SUV total	7.7 (1.55–26.6)	20.8 (5.25–65.65)	0.39		
Affected organs = 1	22 (56.4%)	3 (50%)	0.769		
Affected basin ≤2	29 (70.7%)	5 (83.3%)	0.519		
Ratio SUVmain lesion / SUVmean liver	1.92 (0.52–6.8)	7.67 (0–9.71)	**0.02**	**0.018**	**1.73 (1.09–2.7)**
Ratio SUV total /SUV mean liver	4.1 (0.74–12.85)	13.14 (4.5–39.58)	0.098		

Note: quantitative data are expressed as means and standard deviations or medians and interquartile ranges. Qualitative data are expressed as frequencies and percentages. Only significant results of machine-adjusted p values are shown.

**Fig 2:**
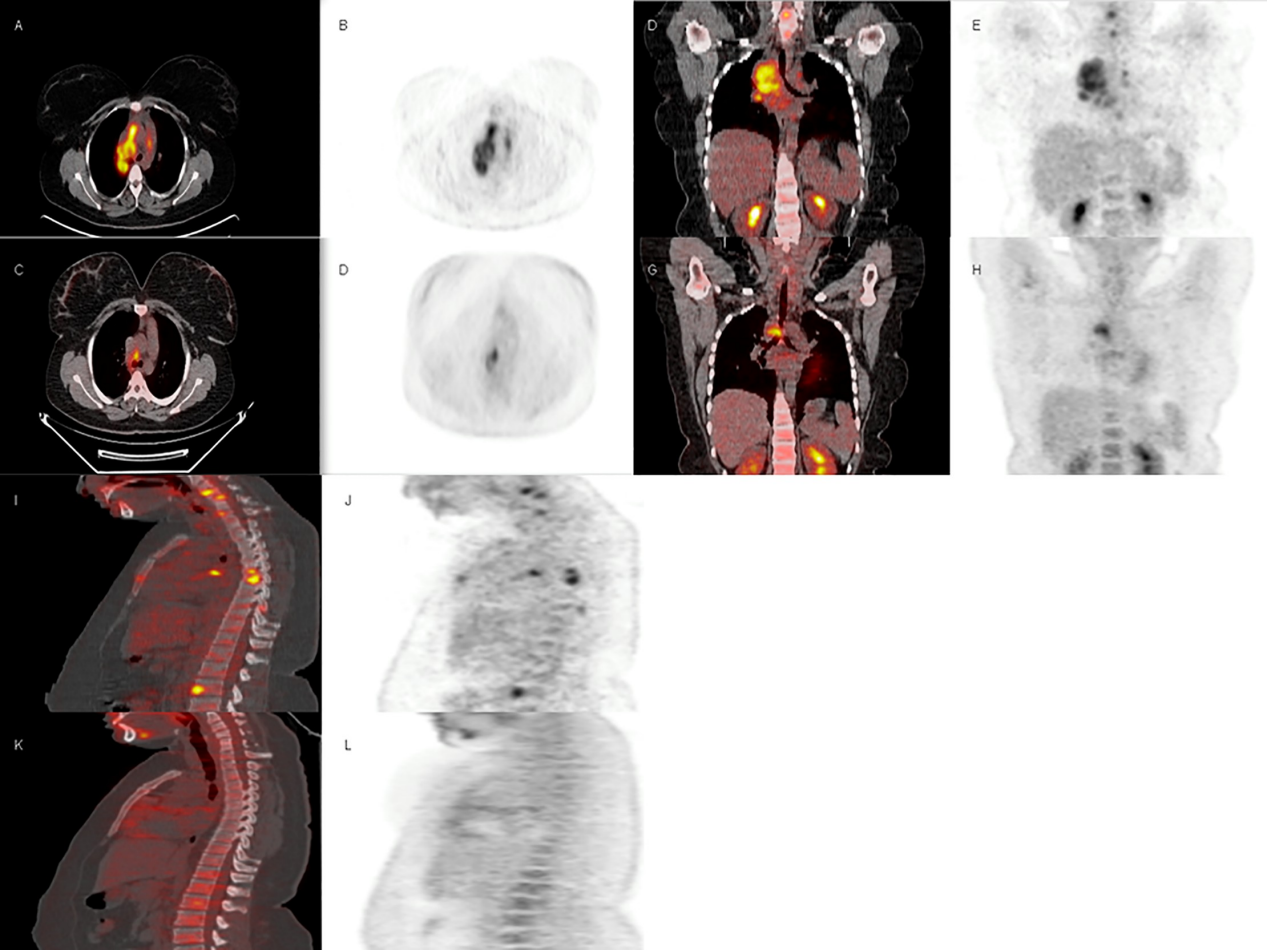
A 20-year-old woman with a mediastinal mass with a necrotizing granulomatosis in the histologic study and a positive culture for *M*. *tuberculosis*. Vertebral lesions were also observed at C5,C6, D5, D6, D7, D11 and L2. A and B (axial plane), E and F (coronal plane), H and I (sagittal plane)) Initial 18F-FDG PET/CT demonstrated an increase uptake in the mediastinic mass with lymph node basin involvement. C and D (axial plane) G and H (coronal), J and K (sagittal plane)) Follow-up 18F-FDG PET/CT after two month of treatment showed reduced 18F-FDG uptake. Concomintant CT showed reduction in the mediastinal mass size and lymph node involvement.

Interestingly, six patients had a follow-up FDG PET/CT (Fig 2; more information in S1 Table). Five out of 6 patients showed improvement or complete resolution in FDG uptake. For the remaining patient, the initial TB lesions resolved completely, however, new uptake regions appeared that were consistent with his polyarteritis nodosa and his newly diagnosed myelodysplastic syndrome.

## Discussion

Our retrospective study assessing the FDG PET/CT in patients with TB diagnosis shows that patients with TB have an increased FDG uptake regardless of the organ involved. FDG PET/TC was often performed in immunosuppressed patients, showing a non-significant trend towards higher FDG uptake at the main lesion than non-immunosuppressed patients. Almost half of the patients had more than one organ affected, and 46.8% of the patients had at least one lymph node basin affected. We found that higher FDG uptake of the main lesion and the lesion size were directly correlated, and that the ratio SUVmain lesion / SUVmean liver may predict successful TB outcome according to WHO criteria.

Pulmonary malignant lesions can be discriminated from benign lesions using FDG PET with high accuracy [15]. Parameters such as uptake rate variation in dual time point evaluation [16], or the use of other tracers may help increase diagnostic accuracy [9]. In TB, FDG PET/CT is useful to differentiate between active and inactive lesions [17]. However, active pulmonary TB lesions may be difficult to distinguish from malignancy [18,19]. Lymph node data from FDG PET/CT have been unable to differentiate between TB-affected and cancer-affected lymph nodes [20]. According to the current knowledge, caution may prevail when distinguishing malignancy from active tuberculosis lesions based on FDG PET/CT regardless of the techniques and tracers used. Histological and microbiologic results must always be sought. Close radiological follow up is recommended after antituberculous treatment even in the presence of *Mycobacterium tuberculosis* in sputum or bronchoscopy samples, given that cancer and tuberculosis may be present together.

We observed a relationship between lesion size and FDG uptake. Consistently, Hara et al noticed that larger TB lesions had a higher FDG accumulation, with similar findings observed in lung cancer [9]. Reasons for this finding may be partially explained by the vascularization and the growth speed. In malignant lesions, small tumours are usually well irrigated due to their proximity to preserved tissue, whereas larger tumours suffer hypoxia in the central part. This increases the glycolysis process, hence increasing the glucose uptake and its analogue FDG. Fast-growing lesions often outrange their oxygen supplies, requiring huge amounts of glucose to maintain their growth rate [9]. The same mechanisms may also explain the higher FDG uptake in bigger TB lesions, along with the activation of monocytes and neutrophils due to the immune response and the mycobacteria replication [21,22]. In animal models, FDG uptake has been associated with the burden of mycobacteria [23,24]. Hence, high uptake of FDG is expected in patients with large and active TB lesions. In our study, we also noticed an association between FDG uptake and the risk of having more than one organ affected and lymph node basin involvement.

CT scan and MRI define TB affected lymph nodes predicated on lymph node enlargement and/or central low attenuation areas indicative of areas of necrosis. FDG PET/CT enables the identification of more affected lymph node basins that otherwise would have gone unnoticed. Interestingly, the number of affected lymph node basins has been correlated with poor response to therapy in patients with non-pulmonary TB and HIV infection, and a cut-off of 5 lymph nodes affected may differentiate responders from non-responders in this subset of patients [20, 25]. While features of a pre-treatment CT scan were unable to predict treatment response in patients with cervical lymphadenitis TB [26]. The lymph nodes coordinate the adaptive immune response against *Mycobacterium tuberculosis* by differentiating T cells into T effector cells. They, in turn, promote macrophage maturation. More affected lymph nodes may suggest a higher effort to control the disease due to a deficient immune system, high *Mycobacterium tuberculosis* burden or a virulent strain.

To date, the burden of TB is poorly defined using conventional test imaging and microbiologic results. FDG PET/CT imaging allows for a better characterization of the burden of disease, suggesting a more extensive involvement than the one defined by other imaging techniques, especially for non-pulmonary TB [25,27]. Shorter drug regimens for TB treatment are urgently needed for both MDR TB and non-MDR tuberculosis, as they will reduce the appearance of resistance due to poor adherence and improve the number of successful outcomes in the programmatic management of TB [28]. To date, clinical trials in susceptible pulmonary TB aiming to demonstrate non-inferiority with shortened TB treatments have failed. On closer analysis, the trials show that a large proportion of patients treated with the short-course treatments had a positive outcome; hence the identification of this subgroup could start a new era for TB. It seems logical to think that patients with a successful TB outcome have a lower burden of disease. FDG PET/CT could quantify the burden of disease by measuring SUVtotal and affected lymph node basins, and identify patients that could benefit from a shortened TB treatment. Nevertheless, a rigorous cost-effectiveness analysis should be undertaken to ensure the viability of this approach.

As treatment response is usually accompanied by a decrease in the lesion size and a decline in inflammation, lower FDG uptake is expected. In a murine model by Davis et al, FDG uptake has shown promising correlation with microbiologic results [29]. FDG uptake reduction may indicate treatment effectiveness and guide the time of the treatment course, mainly in multidrug-resistant (MDR) TB or non-pulmonary TB [25,27]. In our study, follow up FDG PET/CT predicted TB outcome, however the limited number of patients prevent us from drawing any conclusion. A recent study by Malherbe et al prospectively followed patients with pulmonary TB in South Africa and performed FDG PET/CT before, during and after antituberculous therapy and correlated the findings with microbiologic data. According to their results, a non-improved FDG PET/CT result at six months was associated to an unsuccessful TB outcome, although a high proportion of cured patients had ongoing FDG uptake that continues even 1 year after the end of treatment, probably indicating inflammatory activity due to immune response [12]. At the present time, FDG PET/CT may rapidly identify non-treatment responders; however, there are many uncertainties and heterogeneous results that need further investigation.

Our study suffers some limitations inherent to the retrospective nature of the design. Additionally, FDG PET/CT is not programmatically performed in our setting when TB is suspected. Thus, a selection bias is likely to have occurred in our study. As explained above, reasons behind performing the imaging were mainly pulmonary nodules and fever of unknown origin; moreover, our study includes many immunosuppressed patients, who may have an impaired immunity and inflammation activity. A close FDG PET/CT patient follow up was not performed, precluding us from exploring the FDG uptake dynamics in our population. The study encompasses pulmonary TB and non-pulmonary TB which may have differential host-pathogen interactions, thus resulting in FDG uptake heterogeneity. SUV measurements differ between the two scanners used during the study. To homogenize the sample we performed two different approaches as described in the method section. We did not have any treatment failure or recurrence in our patients preventing us from performing any analysis regarding this issue.

In summary, almost half of the patients in our cohort had more than 1 organ affected and 46.8% of them had lymph node involvement. FDG uptake is associated with the size of the lesion in the main organ and it seems to be related to the treatment outcome. The extent of its potential to be used as an early predictor of treatment success still needs to be defined.

## Supporting information

S1 Table FDG PET/CT information of patients with follow-up imaging tests. (DOCX)
