# Supplementary material for: Usefulness of FDG PET/CT in the management of tuberculosis
Source: PLoS One. 2019 Aug 27;14(8):e0221516. doi: 10.1371/journal.pone.0221516 (PMC6711521; doi:10.1371/journal.pone.0221516)
Supplement: S1 Table — (DOCX) [file pone.0221516.s001.docx]

| ID | Sex | Age, y | Immunosuppresion | TB type | Time between PET CT, months | Time diagnosis to control PET TC, months | TB treatment duration | PET CT result | TB result (WHO criteria) |
| --- | --- | --- | --- | --- | --- | --- | --- | --- | --- |
| 1 | F | 20 | None | Disseminated | 2 | 2 (under treatment) | 9 | Improved | Cure |
| 2 | F | 76 | Amyloidosis  Renal failure  Rheumatic polymialgia | Lymph node | 22 | 21 | 7 | Resolved | Cure |
| 3 | M | 76 | Polyarteritis nodosa | Lymph node | 14 | 14 | 12 | Mixed* | Cure |
| 4 | M | 69 | Lung cancer  Hepatic cirrhosis | Lung | 16 | 14 | 6 | Resolved | Cure |
|  |  |  |  |  | 41 | 40 |  | Resolved |  |
| 5 | F | 19 | Renal transplant | Lung | 2 | -1 (treatment was started after the first follow up PET TC) | 8 | Mixed | Cure |
|  |  |  |  |  | 35 | 32 |  | Resolved |  |
| 6 | M | 60 | Esophagus cancer | Lung | 13 | 10 | 6 | Mixed† | Cure |
|  |  |  |  |  | 20 | 17 |  | Mixed† |  |

S1 Table. Data from patients with follow up imaging.

ID: identificator; TB: tuberculosis, PET-CT: positron emission tomography-computed tomography; WHO: world health organization. Improved: reduction in the size and SUV uptake of the TB lesions. Mixed: improvement of some lesions, but worsening of others or appearance of new lesion. Worsened: increase in the size or SUV of TB lesions.

*Patient with Polyarteritis nodosa with poor response to treatment and new myelodisplastic syndrome. Tuberculosis lesions resolved.

†Esophagus cancer progression (Histologically confirmed). Tuberculosis lesion improved.
